# Cerebrovascular Compliance Within the Rigid Confines of the Skull

**DOI:** 10.3389/fphys.2018.00940

**Published:** 2018-07-17

**Authors:** Mair Zamir, M. Erin Moir, Stephen A. Klassen, Christopher S. Balestrini, J. Kevin Shoemaker

**Affiliations:** ^1^Department of Applied Mathematics, The University of Western Ontario, London, ON, Canada; ^2^Department of Medical Biophysics, The University of Western Ontario, London, ON, Canada; ^3^School of Kinesiology, The University of Western Ontario, London, ON, Canada; ^4^Department of Physiology and Pharmacology, The University of Western Ontario, London, ON, Canada

**Keywords:** cerebral blood flow, cerebrovascular compliance, intracranial compliance, intracranial pressure, pulsatile blood flow

## Abstract

Pulsatile blood flow is generally mediated by the compliance of blood vessels whereby they distend locally and momentarily to accommodate the passage of the pressure wave. This freedom of the blood vessels to exercise their compliance may be suppressed within the confines of the rigid skull. The effect of this on the mechanics of pulsatile blood flow within the cerebral circulation is not known, and the situation is compounded by experimental access difficulties. We present an approach which we have developed to overcome these difficulties in a study of the mechanics of pulsatile cerebral blood flow. The main finding is that while the innate compliance of cerebral vessels is indeed suppressed within the confines of the skull, this is compensated somewhat by compliance provided by other “extravascular” elements within the skull. The net result is what we have termed “intracranial compliance,” which we argue is more pertinent to the mechanics of pulsatile cerebral blood flow than is intracranial pressure.

## 1. Introduction

**Figure 1:**
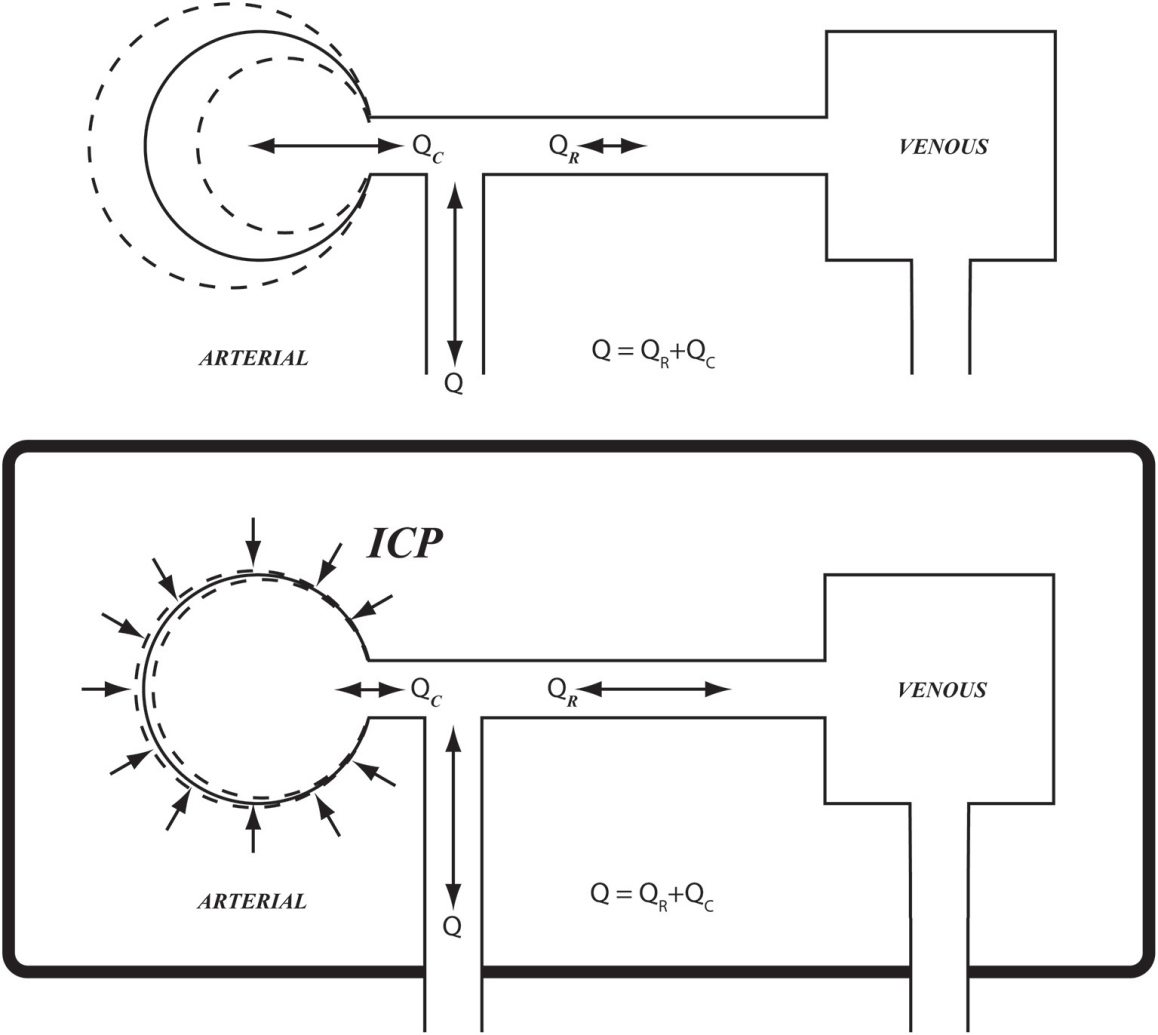
Schematic illustration of arterial compliance as it is constrained within the confines of the rigid skull **(Bottom)**, compared with arterial compliance in the vascular bed of the forearm where it is relatively free from such constraint **(Top)**. *ICP*, intracranial pressure; *Q*, total flow rate going into the cerebral circulation; *Q*_*R*_, *Q*_*C*_, resistive and capacitive components of flow rate, respectively.

Under conditions of pulsatile blood flow, blood vessels exercise their compliance to absorb the rising pressure in systole by simply distending locally and momentarily to accommodate the passage of the pressure wave (Lighthill, 1975; London and Guerin, 1999; Zamir, 2016). This scenario is possible in most regions of the cardiovascular system because the vessels can distend locally, relatively free from external constraints. This is not so in the case of cerebral blood flow, however, because of the constraints of the rigid skull (Figure 1). The effects of these constraints on the pulsatile dynamics of cerebral blood flow are not known. As a result, a clear interpretation of pressure and flow measurements within the cerebral circulation, or indeed of any other oscillatory phenomenon within the rigid confines of the skull, is currently unattainable.

The study of cerebral blood flow has in general been a challenge because of access difficulties for obtaining direct intracranial measurements (Purves, 1978; Weyland et al., 2000; Grune and Klimek, 2017; Weiland et al., 2017). While the study of perfusion of specific regions of the brain has made remarkable strides with the advent of magnetic resonance imaging (MRI) technology, the study of pressure and flow distributions within cerebral vasculature remains a challenge. In particular, measurements of pressure and flow waveforms within the cerebral vascular bed, which would allow an exploration of the mechanics of pulsatile blood flow within that bed, have not been available so far. An understanding of these mechanics cannot be determined from measures of localized perfusion of brain tissue obtained by MRI because perfusion is the end product of these mechanics. The means by which this end product is achieved requires a study of the mechanics of flow within the cerebral vasculature, which involves the relationship between beat-to-beat pressure and flow waveforms. The latter is beyond the speed capability of MRI. In this paper we present an approach which we have developed specifically for this purpose.

Earlier studies of cerebral blood flow relied on observable changes in pial artery diameter, with the view that an increase or a decrease in vessel diameter indicates an increase or a decrease in flow, respectively (Lassen, 1959; Purves, 1978). However, while a change in vessel diameter is indeed one the main mechanisms for producing a change in flow rate in the cardiovascular system; it is not the only mechanism because it affects only the steady (mean) component of pulsatile blood flow. The oscillatory component of pulsatile blood flow within a blood vessel or within a vascular bed as a whole is affected by the compliance and viscoelasticity of the vessel walls as well as the inertial environment within the vessel or vascular bed. Indeed, the mathematical relationship between the oscillatory pressure and the oscillatory flow rate in a vascular bed, and the way this relationship depends on the mechanical properties of that bed, is well established (Zamir, 2005). Thus, given the mechanical properties of the vascular bed, it is possible to predict the flow waveform that results from a given driving pressure waveform. Conversely, and perhaps more importantly, given simultaneous measurements of pressure and flow waveforms at the entrance to a vascular bed, the relationship between them can be invoked to determine the mechanical properties of that bed.

The above approach provides a non-invasive tool for determining the mechanical state and properties of a vascular bed and is therefore particularly pertinent to a study of the cerebral vascular bed because of access difficulties to that bed. We have used this tool successfully in the past to explore the vascular bed of the lower arm where simultaneous measurements of oscillatory pressure and flow can be made via the brachial artery (Zamir et al., 2009). Use of the same techniques to study the cerebral vascular bed faces two main difficulties. First, simultaneous measurements of oscillatory pressure and flow waveforms within the cerebral circulation are currently not possible. Second, the effect of intracranial constraints on the relationship between the oscillatory pressure and flow is not known. In this paper we report the results of an approach which we have used to address these challenges in a study of the pulsatile dynamics of cerebral blood flow. Our specific aims were to (a) establish the utility of systemic pressure waveforms in the analysis of cerebral blood flow, and (b) derive a measure of cerebrovascular compliance within the rigid confines of the skull.

## 2. Methods: experiment

### 2.1. Participants

Six young healthy adults (24 ± 3 years; mean ± SD; range 21–29 years; 5 females) participated in the current investigation. Participants were 166 ± 6 cm in height and 61 ± 5 kg in weight (BMI 22 ± 2 kg/m^2^), recreationally active and were non-smokers with no history of cardiovascular disease. All participants provided written, informed consent and the study was approved by the Health Sciences Research Ethics Board at Western University.

### 2.2. Experimental protocol

Participants rested in a supine position during instrumentation. A standard 3-lead electrocardiogram (ECG) provided continuous measures of heart rate (HR). Beat-by-beat measures of arterial blood pressure (ABP) waveforms were collected at the left middle or index finger and transformed to brachial artery pressure waveforms using a finger photoplethysmograph with a built in transfer function (Finometer, Finapres Medical Systems, Amsterdam, The Netherlands). Transcranial Doppler Ultrasound (TCD) (Neurovision 500M, Neurovision TOC2M Multigon Industries, Elmsford, CA, USA) provided flow velocity waveforms from the left middle cerebral artery (MCA) (2 MHz PW Doppler probe). This waveform represented the instantaneous peak cerebral blood flow velocity (CBFV). The blood velocity waveforms of the right brachial artery were collected using Duplex ultrasound (10-MHz probe; Vivid 7 system; GE Healthcare Canada, Mississauga, ON, Canada). These waveforms reflected the instantaneous mean flow velocity using a window that captured the luminal width of the artery. Following stabilization of hemodynamic parameters, one to 2 min of supine data were collected simultaneously. All analog signals were sampled in real time at 1,000 Hz with an online acquisition and analysis system (PowerLab, ADInstruments; Castle Hill, New South Wales, Australia) and stored digitally for subsequent analysis.

### 2.3. Data analysis

The brachial blood pressure waveform obtained by the Finometer device was corrected to manual values. Pressure and flow velocity waveforms were recorded continuously during the experiment. Five waveforms for computing hemodynamic properties of the brachial and middle cerebral vascular beds, as described below, were selected from a stable segment of the record.

## 3. Methods: theory

### 3.1. Background

The relationship between the oscillatory components of pressure and flow in pulsatile blood flow depends on four main properties of the vascular bed in which pressure and flow are being measured, namely the total resistance *R* to the steady component of flow in that bed, the collective (“lumped”) compliance *C* of the vessels comprising the bed, the viscoelastic resistance to stretch *K* within the vessel walls, and the prevailing inertial effects *L* of accelerating/decelerating flow within the vascular bed as a whole (Zamir, 2005).

These four properties are useful markers of the mechanical/functional health of a vascular bed because they determine the efficiency of oscillatory dynamics of blood flow within the blood vessels comprising that bed. In particular, they determine the exact flow waveform produced by a given pressure waveform driving the flow in that bed.

The relationship between the pressure and flow waveforms in a vascular bed is generally based on the mathematical relationship between individual harmonics (*p, q*) of the pressure and flow waveforms, namely (Zamir, 2005)

(1) $$\begin{array}{l} q=\frac{p}{Z} \end{array}$$

where “*Z*” is the impedance of the vascular bed, given by

(2) $$\begin{array}{l} Z=\frac{R\left(\omega KC+i\left(\omega^{2}LC-1\right)\right)}{\omega C\left(K+R\right)+i\left(\omega^{2}LC-1\right)} \end{array}$$

*R, C, K, L* are the properties of the bed as described above, ω is the oscillatory frequency and $i=\sqrt{-1}$. Thus, if pressure and flow waveforms are measured, the above relationship between their individual harmonics can be used to compute the values of the properties *R, C, K, L*.

**Figure 2:**
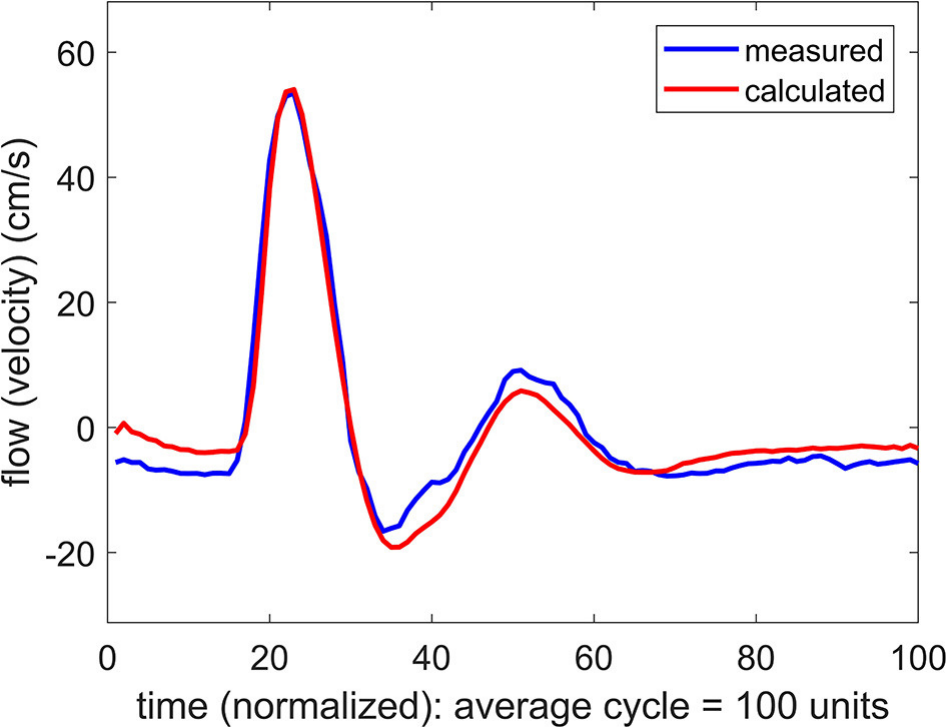
Comparison of measured and calculated flow (velocity) waveforms in the vascular bed of the forearm, based on a pressure waveform measured simultaneously with the flow waveform at the brachial artery.

Briefly, the value of the resistance *R* is first determined from

(3) $$\begin{array}{l} R=\frac{\bar{P}}{\bar{Q}} \end{array}$$

where $\bar{P}$ and $\bar{Q}$ are mean pressure and mean flow, respectively, over the oscillatory cycle. Then, initial parameter values *C*_0_, *K*_0_, *L*_0_ are prescribed and a flow waveform is determined based on these values, using Equations 1, 2. This “predicted” flow waveform is compared with the *measured* flow waveform and the difference between the two is calculated as an error *E* defined by

(4) $$\begin{array}{l} E=\frac{1}{{\bar{Q}}^{2}}\overset{100}{\underset{j=1}{\sum }}\Delta q_{j}^{2} \end{array}$$

where Δ*q*_*j*_ is the difference between the predicted and the measured data points at time instant *j*(= 1⋯100) within the oscillatory cycle. An iterative scheme is then established to reduce the value of *E* by changing the values of the parameters *C, K, L* from the initially prescribed values *C*_0_, *K*_0_, *L*_0_ in a systematic process. First, while holding the values of *K*(= *K*_0_) and *L*(= *L*_0_), the value of *C* is changed to a range of values above and below *C*_0_ to find a value *C* = *C*_1_ for which the error *E* is a minimum. Then, while holding the values of *C*(= *C*_1_) and *L*(= *L*_0_), the value of *K* is changed to a range of values above and below *K*_0_ to find a value *K* = *K*_1_ for which the error *E* is a minimum, and a value *L* = *L*_1_ is then found in a similar way. The entire process is then repeated for another iteration to find *C*_2_, *K*_2_, *L*_2_, and another to find *C*_3_, *K*_3_, *L*_3_ etc. In each iteration the minimum value of *E* is found to higher accuracy, and the process ends when a prescribed accuracy is reached. The process is tedious but is automated within a computational scheme. The ultimate values of *C, K, L* at which the process ends are then deemed to be representative of the corresponding properties of the vascular bed. An example of the result is shown in Figure 2.

### 3.2. Application to the cerebral vascular bed

A key requirement for the use of the above approach is the simultaneous measurement of pressure and flow waveforms at entry to the vascular bed under investigation. At present this requirement cannot be met in the cerebral vascular bed because of access difficulties. In this study we explored the use of systemic pressure waveforms measured simultaneously with cerebral flow waveforms. Thus an additional aim of our study was to examine the validity of this compromise as a tool to explore the mechanics of pulsatile blood flow within the confines of the rigid skull.

## 4. Results

### 4.1. Use of systemic blood pressure as a substitute for cerebral blood pressure

**Figure 3:**
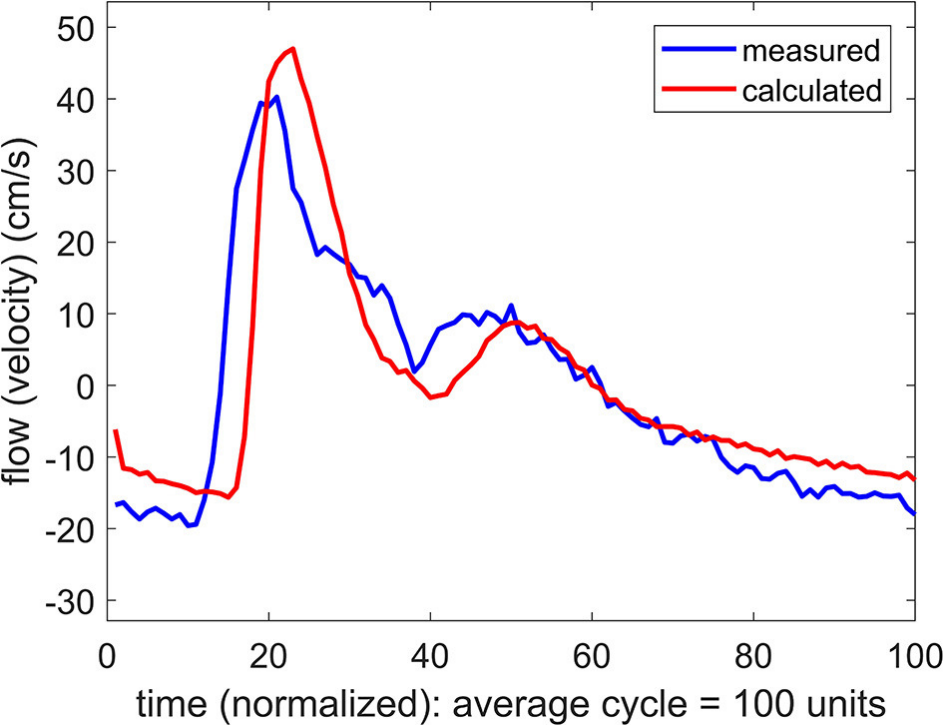
Comparison of measured and calculated flow (velocity) waveforms in the cerebral vascular bed, the latter being based on *systemic pressure* waveform measured simultaneously at the brachial artery.

The result of using a systemic pressure waveform in combination with a simultaneously measured flow (velocity) waveform at the MCA is shown in Figure 3. In this process, as for the results in Figure 2, Equations 1, 2 are used to predict a flow waveform to be compared with the measured flow waveform. The values of the parameters *R, C, K, L* are adjusted in an iterative manner to achieve the closest agreement between the predicted and the measured flow waveforms.

The difference between the measured and the predicted flow waveforms observed in Figure 3 clearly arises because the pressure waveform on which the calculated flow waveform is based is the systemic, not the (unknown) cerebral, pressure waveform. It was therefore important to determine if the difference between these two pressure waveforms is a difference in form, or only in phase as the results in Figure 3 strongly suggest.

If the difference between the systemic pressure waveform and the unknown cerebral pressure waveform is a difference in the form of the two waves, then the predicted flow waveform based on this substitution is not valid because it would be based on values of the properties *R, C, K, L* which do not represent the actual values of these properties within the cerebral circulation. To test this possibility we applied changes to the values of *R, C, K, L* in an iterative attempt to close the gap between the two flow waveforms in Figure 3. Using the same iterative scheme for this purpose as was used in Figure 2, we found that it was not possible to bring the two waveforms closer together by systematic changes in the values of *R, C, K, L*.

**Figure 4:**
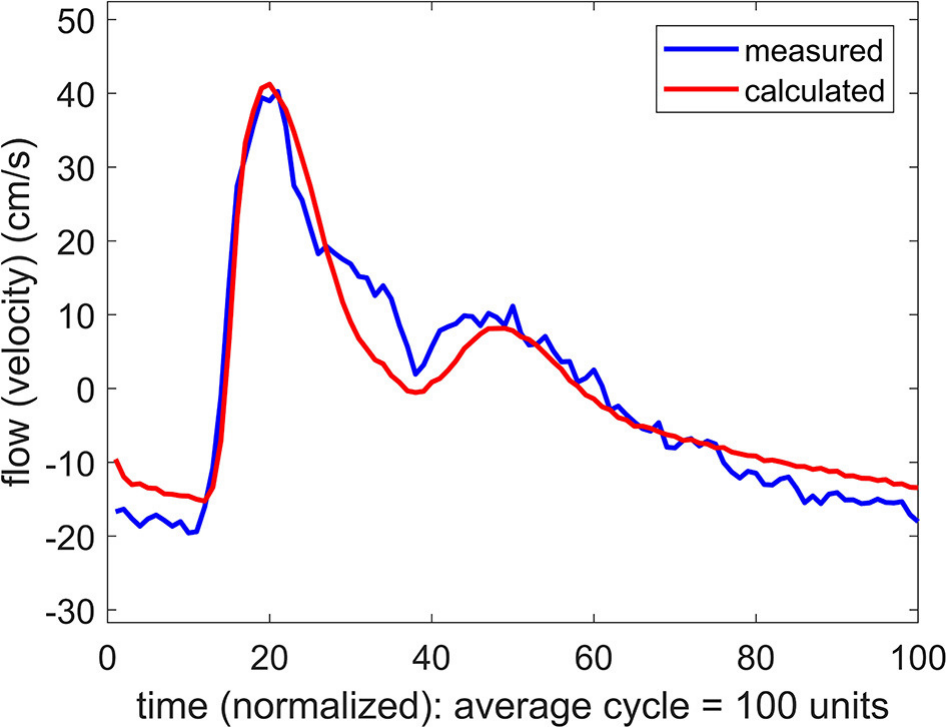
Comparison of measured and calculated flow (velocity) waveforms in the cerebral vascular bed, the latter being based on *systemic pressure* waveform measured simultaneously at the brachial artery, as in Figure 3, but here the pressure is shifted in time by 40 ms.

If the difference between the systemic pressure waveform and the unknown cerebral pressure waveform is only a difference in phase, with a time shift of say “Δ*t*,” then the systemic pressure waveform can be used as a substitute for the cerebral pressure waveform by applying a simple time shift “Δ*t*” to the systemic pressure waveform. This modified pressure waveform can then be used to predict a cerebral flow waveform to be compared with the measured cerebral flow waveform. The result of one such substitution is shown in Figure 4 where a remarkable agreement is seen between the measured and the predicted flow waveforms.

The time shift “Δ*t*” represents the difference between (a) the time it takes the pressure pulse to travel from the aorta to the brachial artery where it is measured, and (b) the time it takes the same pressure pulse to travel to the MCA where it is assumed to arrive on the basis of the measured flow wave. Since pulse wave velocity depends generally on vessel diameter as well as on vessel wall properties, the magnitude of the time shift “Δ*t*” will depend not only on the distance traveled in each case but also on the nature of the vascular path being traveled within the vascular bed. It is clear then that the magnitude of “Δ*t*” will be specific to each subject because of differences in vascular branching architecture. This is indeed what we found: Values of “Δ*t*” for the six subjects ranged from 25 to 50 ms.

On the basis of these results we concluded that systemic pressure waveforms, with appropriate time shift, can be used as a substitute for the unknown cerebral pressure waveforms in the relationship between pressure and flow in Equations 1, 2 to study the dynamics of pulsatile blood flow within the cerebral circulation. The time shift Δ*t* was determined individually for each subject as described above.

### 4.2. Cerebrovascular compliance

In each of six subjects, compliance of the cerebrovascular bed was determined, using the above methodology. For the purpose of comparison with a vascular bed with minimal extravascular pressure constraints and with known properties from earlier studies, compliance of the vascular bed of the forearm was also determined simultaneously for each subject. In both cases, because of the instrumentation involved (TCD in the brain, ultrasound in the arm), the magnitude of the compliance *C* was based on velocity rather than flow waveforms. This produced a scaled value of *C*, the scaling being different in the two cases because it depended on the position within the vessel lumen where the velocity was being measured.

Thus, while the scaled values of *C* in the arm and in the brain could not be compared directly, the main aims of our study were (a) the ability to detect cerebrovascular compliance within the confines of the rigid skull using this methodology and (b) to compare the nature and behavior of this compliance with vascular compliance in the vascular bed of the forearm.

### 4.3. Intracranial compliance (*ICC*)

**Figure 5:**
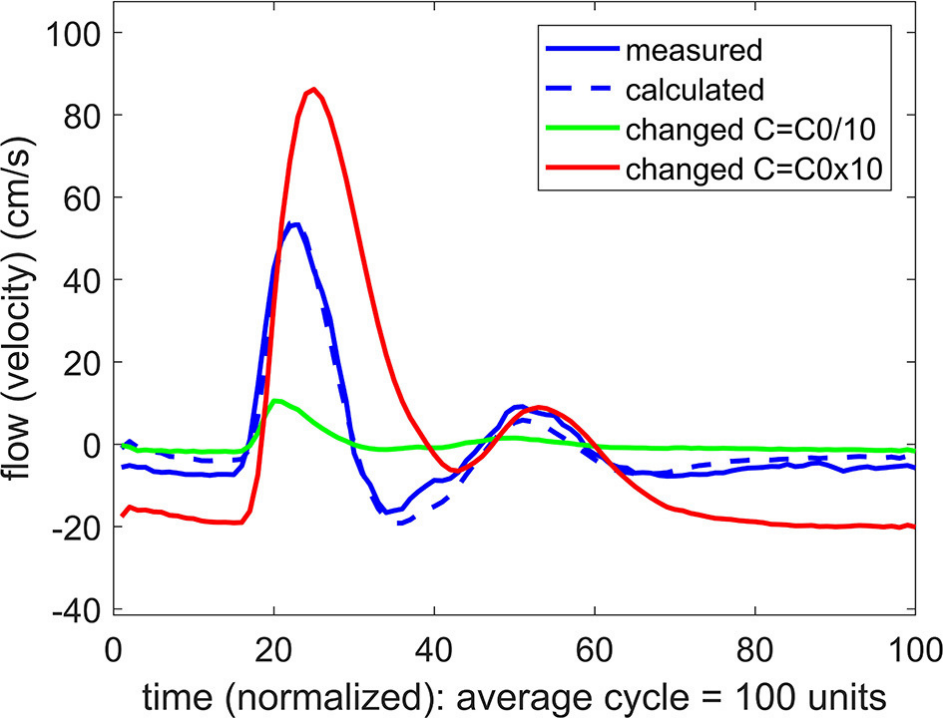
A test of the role of compliance “*C*” in the relationship between pulsatile pressure and flow (velocity) in pulsatile blood flow within the vascular bed of the forearm. The actual compliance “*C*_0_” of the vascular bed is determined by agreement between the measured and predicted flow waveform. When this value of *C* is changed to either higher or lower values, the predicted flow (velocity) waveform becomes vastly different from the measured flow waveform, indicating that compliance is playing a significant role in the relationship between pulsatile pressure and flow.

**Figure 6:**
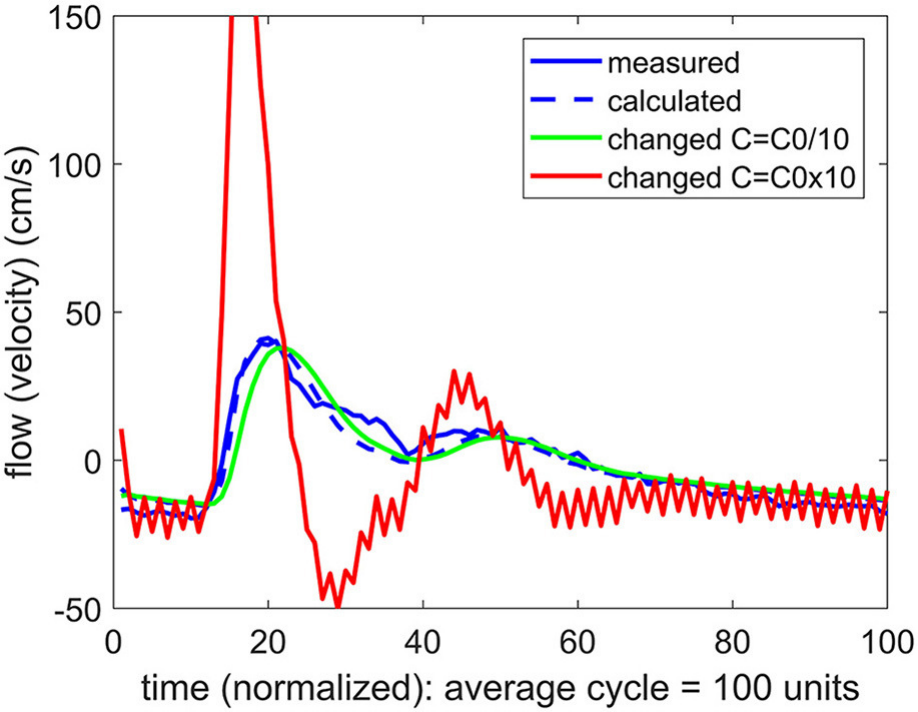
A test of the role of compliance “*C*” in the relationship between pulsatile pressure and flow (velocity), as in Figure 5, but here applied to pulsatile blood flow within the cerebral vascular bed. The actual compliance “*C*_0_” of the vascular bed is here again determined by agreement between the measured and predicted flow waveform. When this value of *C* is changed, however, the result depends on whether the change is to higher or to lower values. At lower values of *C* there is little effect, indicating that compliance is not playing a significant role in the relationship between pulsatile pressure and flow.

To pursue the aim in (b) above, we examined the effect of a change in the value of the compliance *C* on the form of the calculated flow wave. More precisely, if agreement between the measured and the predicted flow waveforms was attained at a value of *C* = *C*_0_, two new flow waveforms were calculated, one with a higher value of *C* than *C*_0_, and one with a lower value. In the vascular bed of the forearm, the change in the value of *C* generally produced a large change in the flow waveform, whether the change was to higher or to lower values of *C*, as shown in Figure 5. In the brain, however, the same exercise produced a different result. Here a change in the value of *C* only to higher values produced a large change in the flow waveform. A change to lower values of *C* had comparatively little or no effect, as shown in Figure 6.

To quantify the difference between the results in Figures 5, 6, we used the error *E* as defined in Equation 4. To normalize the effect of a change in the value of *C* on the flow waveform, a normalized form of this error was then defined by

(5) $$\begin{array}{l} EE=\frac{E}{E_{0}} \end{array}$$

where *E*_0_ is the error between the measured and the predicted flow waveforms with *C* = *C*_0_, and *E* is the corresponding error with values of *C* higher or lower than *C*_0_.

To facilitate comparison between the results for the cerebrovascular bed with that of the forearm, the changed compliance *C* was scaled in each case by the baseline compliance *C*_0_ and a scaled measure of compliance was defined by

(6) $$\begin{array}{l} CC=\frac{C}{C_{0}} \end{array}$$

**Figure 7:**
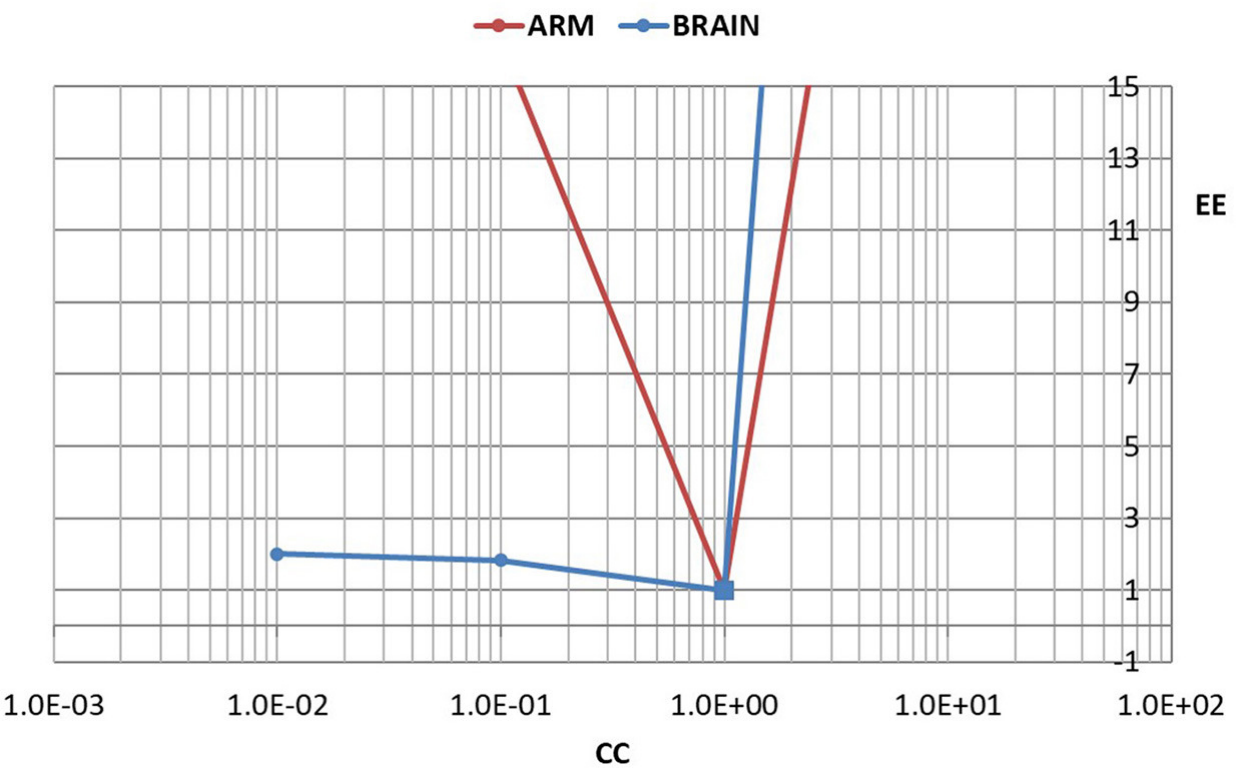
A test of the role of compliance in the relationship between pulsatile pressure and flow, as in Figures 5, 6, but here the effect of a change in compliance is quantified in terms of a normalized error “*EE*” between the measured and predicted flow waveform as defined in the text. The error is scaled to 1.0 when there is agreement between the two waveforms (square markers). To facilitate comparison between the results for the cerebrovascular bed with that of the forearm, the changed compliance was scaled in each case by the baseline compliance and a scaled measure of compliance “*CC*” is shown, as defined in the text. In the arm, a change in the value of “*CC*” to either higher or lower values is seen to lead to large errors. In the brain, by contrast, a change to lower values of “*CC*” leads to relatively small error.

The results are shown in Figure 7.

## 5. Discussion and conclusions

Improved understanding of the mechanics of oscillatory cerebral flow requires simultaneous measurements of oscillatory beat-to-beat pressure and flow within the cerebral circulation. While TCD provides some access to flow (velocity) measurements, no such access exists for the corresponding measurements of pressure. The lack of access to pulsatile pressure measurements within the cerebral circulation is in fact particularly critical because of the unknown effects of intracranial pressure (ICP) on the mechanics of this circulation.

In this study we explored the use of systemic blood pressure as a substitute for cerebral blood pressure. The success of this substitution depends on the integrity of the oscillatory pressure waveform as it travels from the brachial artery, where it was measured, to the MCA. While we have no measurement of the form of the pressure wave as it arrives at the MCA, we are able to infer that form from the corresponding flow waveform which was measured at that location. The results in Figures 2, 3 indicate, as would be expected, that there is a phase difference (time shift) between the pressure waveform as it is measured in the arm and as it reaches its destination in the cerebral vascular bed (where its form is inferred from the measured flow (velocity) waveform). More significantly, however, the results in Figure 3 indicate that the form of the wave remains largely intact during that time. The results were fairly uniform across subjects, with only an expected difference in the magnitude of the time shift between subjects because of differences in their size and vascular bed architecture. Thus, overall, we found that the results in the six subjects we used provided a reasonable basis for using systemic blood pressure as a substitute for cerebral blood pressure, thereby providing a non-invasive methodology to explore the mechanics of pulsatile blood flow within the constraints of the rigid skull and, in particular, to explore the “effective compliance” of the cerebrovascular bed within these confines.

Our most important finding in this exploration is embodied in the results of Figures 5–7. Our interpretation of these results is that the mechanics of pulsatile blood flow within the rigid confines of the skull is governed not only by the intrinsic compliance of the cerebral vasculature but also by other sources of compliance within the skull. The concept of extravascular compliance has been suggested indirectly by other authors in the past (Greitz, 1993; Mase et al., 1998; Bateman, 2000; Egnor et al., 2002; Portella et al., 2005; Bateman et al., 2008; Warnert et al., 2016). We believe that our results provide strong supportive evidence for this concept. Among possible sources of extravascular compliance within the skull is the compliance of brain tissue and the virtual compliance provided by changes in volume of venous blood and cerebrospinal fluid within the skull.

Thus the “effective compliance” of the cerebral vascular bed is a combination of intravascular and extravascular compliance which we referred to as intracranial compliance or *ICC*. This combination is more pertinent to the dynamics of pulsatile blood flow within the cerebral circulation than is *ICP*. While *ICP* is widely considered as an important clinical marker, often in relation to hydrocephalus, autoregulation, or zero-flow-pressure, and while it does indeed have effects on brain tissue and on the volume of and movement of spinal fluid and venous blood within the skull, these effects are not fully understood because they are neither direct nor readily measurable (Matsumoto et al., 1986; Ursino et al., 1998; Czosnyka and Pickard, 2004; Portella et al., 2005; Wilson et al., 2005; Kashif et al., 2012; Wilkie et al., 2012; Grune et al., 2015; Winklewski et al., 2015; de Jong et al., 2017; Oswal and Toma, 2017; Rajesh et al., 2017; Tymko and Ainslie, 2017; Gruszecki et al., 2018). By contrast, our results demonstrate that *ICC* has a clear interpretation and can be measured directly and noninvasively. More specifically, it is the value of ‘*C*’ associated with Figure 3, obtained by using Equations 1, 2 in conjunction with the measured flow waveform at the MCA. Furthermore, in combination with the mathematical relationship between oscillatory pressure and flow within the cerebral circulation, *ICC* can be used as a tool to probe into the mechanical environment within the confines of the skull as illustrated in what follows.

In the absence of external constraint, the relationship between pressure and flow in Equations 1, 2 would indicate the presence of a level of compliance “C = C_0_” under which there is agreement between the measured and the calculated flow (velocity) waveform as shown in Figure 2. That *C*_0_ is indeed a measure of the true compliance of the forearm vasculature is supported by the results in Figure 5. Here a change in this value of *C* to values higher or lower than *C*_0_ is seen to cause complete departure from the agreement between the measured and calculated flow waveforms. A repeat of this test in the cerebral circulation, however, produced different results, as shown in Figure 6. Here a change in the value of *C* led to different results depending on whether the change was to values higher or to lower than *C*_0_. A change to a value higher than *C*_0_ causes complete departure from the agreement between the measured and calculated waveform, as in the forearm. However, a change to a value lower than *C*_0_ has a much smaller effect. This difference between the cerebral and forearm results is shown more succinctly in Figure 7, in terms of the error produced by the change in the value of *C*.

From a mathematical perspective, if a change in the value of *C* in Equations 1, 2 produces no change in the predicted flow (velocity) waveform, then the compliance *C* as a variable is absent from the relationship between oscillatory pressure and flow in the system represented by these equations. Conversely, if a change in the value of *C* in Equations 1, 2 produces a large change in the predicted flow waveform, then compliance *C* is very much at play in the relationship between oscillatory pressure and flow in the system represented by these equations.

**Figure 8:**
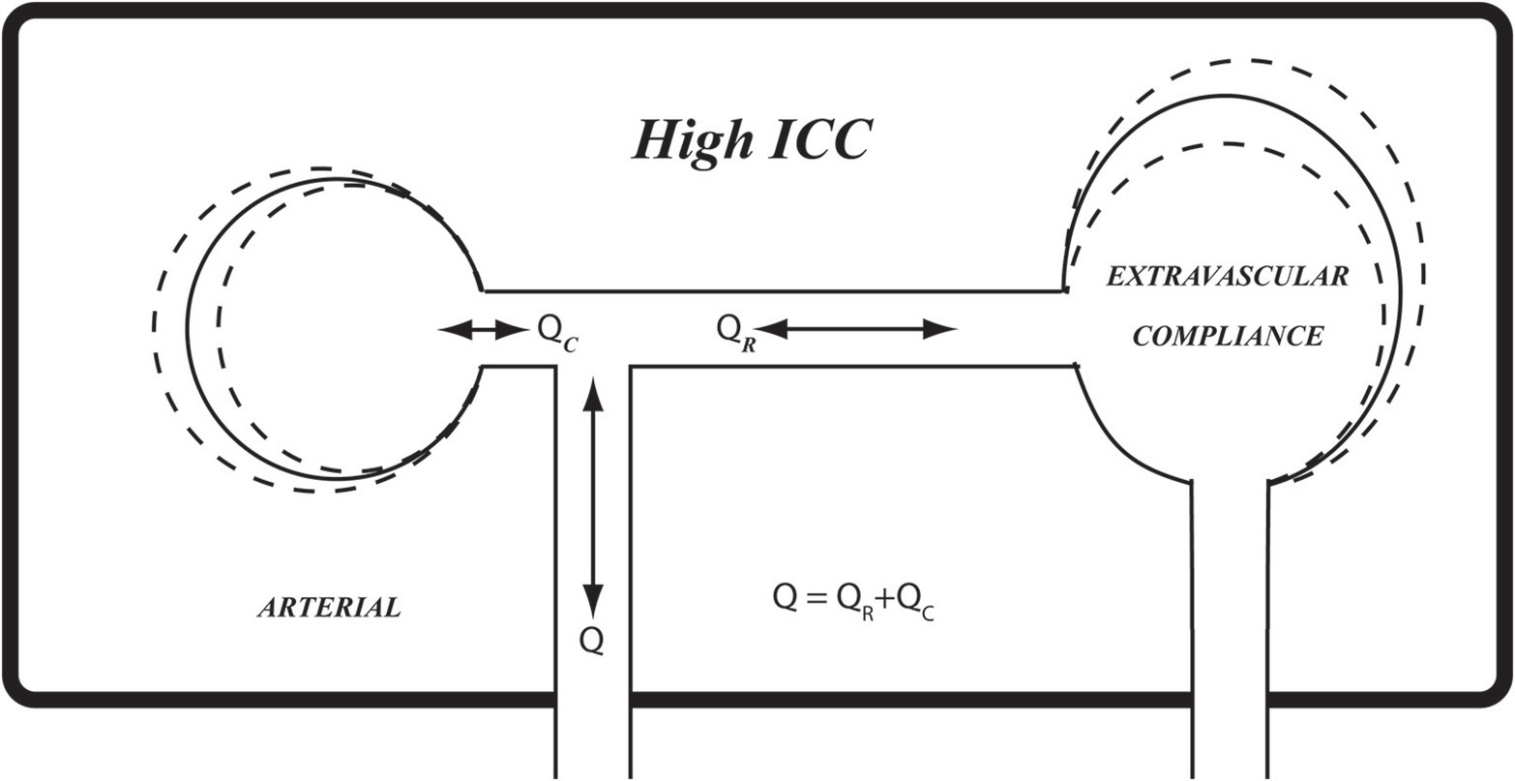
A schematic representation of extravascular compliance within the rigid skull, as discussed in the text. While compliance of cerebral vasculature is constrained within the rigid skull, compliance of other elements within the skull, such as soft brain tissue, venous and spinal fluid volumes, provide extravascular sources of compliance. The combination of prevailing intravascular and extravascular compliance is what we referred to as intracranial compliance (*ICC*) as distinct from intracranial pressure (*ICP*). The relationship between pulsatile pressure and flow within the skull is governed by *ICC* rather than by compliance of the cerebral vascular bed. High *ICC*, as shown here schematically, may be likened by analogy to fully inflated balloon.

**Figure 9:**
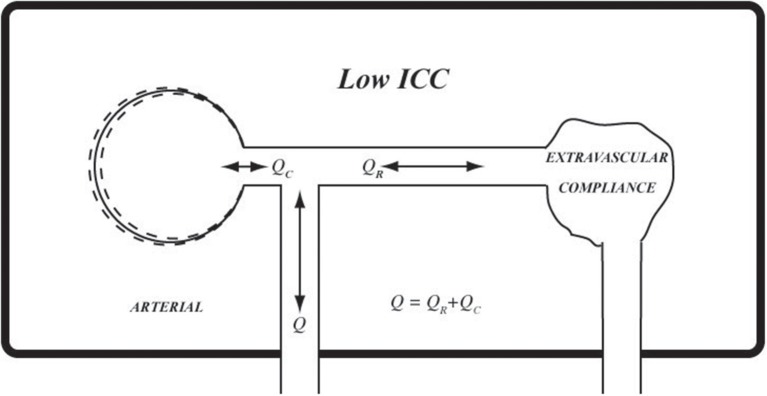
Using the same analogy as in Figure 8, at low *ICC* extravascular compliance may be likened to a deflated balloon which provides very little or no extravascular compliance.

**Figure 10:**
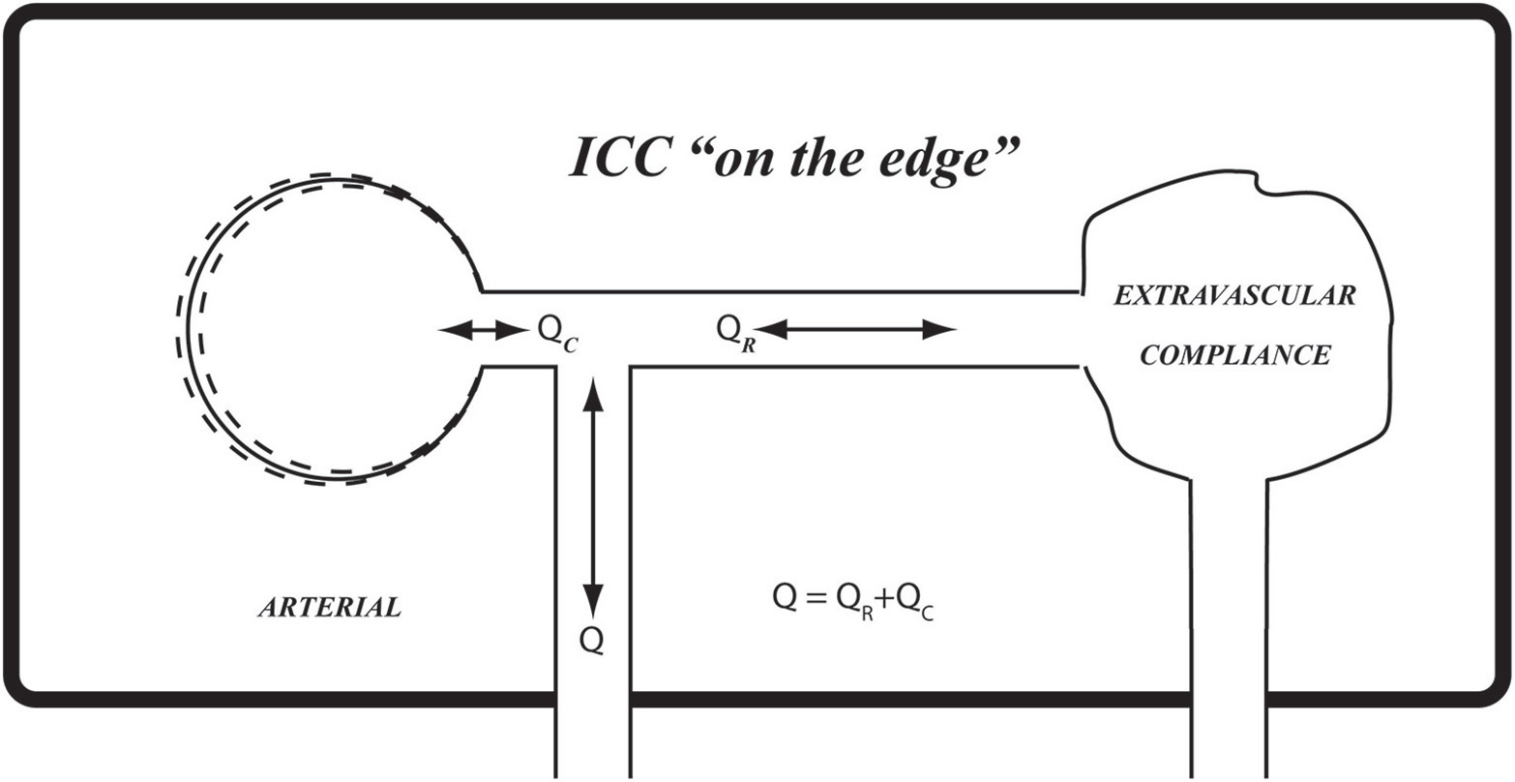
The results in Figures 6, 7 suggest that the prevailing compliance for pulsatile blood flow within the confines of the rigid skull (*ICC*) is at a critical value which we have termed “on the edge.” Using the balloon analogy, it can be likened to a balloon that is inflated to a critical volume whereby it provides extravascular compliance if inflated any further and no compliance if it is deflated any further.

Our interpretation of the results in Figures 5–7 is that the level of *ICC* under which pulsatile blood flow occurs within the cerebral circulation is “on the edge,” or at a critical value (*ICC*_0_) such that when *ICC*>*ICC*_0_, there is some effective compliance for the cerebral circulation, but when *ICC*<*ICC*_0_, the cerebral circulation operates with very little or no effective compliance. These scenarios are illustrated schematically in Figures 8–10, using the analogy of an inflated balloon to represent the level of extravascular compliance within the skull. Since the latter is suspected to involve the inflow and outflow of intracranial venous blood and spinal fluid, and possibly the compliance of brain tissue, the monitoring and measurement of *ICC* as demonstrated in this study provide a useful “window” into the mechanical state of pulsatile blood flow within the cerebral circulation.

## Ethics statement

All participants provided written, informed consent and the study was approved by the Health Sciences Research Ethics Board at the University of Western Ontario.

## Author contributions

MZ, JS, and MM conception and design of the study. MM, SK, and CB data acquisition. MZ drafting. MZ, JS, MM, SK, and CB revising. All authors provide approval for publication and agree to be accountable for all aspects of the work.

### Conflict of interest statement

The authors declare that the research was conducted in the absence of any commercial or financial relationships that could be construed as a potential conflict of interest.
